# Comparative Microbiome Profiles of Sympatric Tick Species from the Far-Western United States

**DOI:** 10.3390/insects10100353

**Published:** 2019-10-18

**Authors:** Betsabel Chicana, Lisa I. Couper, Jessica Y. Kwan, Enxhi Tahiraj, Andrea Swei

**Affiliations:** 1 Quantitative and Systems Biology Program, University of California, Merced, CA 95343, USA; bchicanaromero@ucmerced.edu; 2 Department of Biology, Stanford University, Palo Alto, CA 94305, USA; lisabelcouper@gmail.com; 3 School of Veterinary Medicine, University of California, Davis, CA 95616, USA; jessykwan912@gmail.com; 4 Department of Biology, San Francisco State University, 1600 Holloway Ave, San Francisco, CA 94132, USA

**Keywords:** tick, microbiome, endosymbiont, *Ixodes*, *Dermacentor*, *Haemaphysalis*

## Abstract

Insight into the composition and function of the tick microbiome has expanded considerably in recent years. Thus far, tick microbiome studies have focused on species and life stages that are responsible for transmitting disease. In this study we conducted extensive field sampling of six tick species in the far-western United States to comparatively examine the microbial composition of sympatric tick species: *Ixodes pacificus*, *Ixodes* *angustus*, *Dermacentor variabilis*, *Dermacentor occidentalis*, *Dermacentor albipictus*, and *Haemaphysalis leporispalustris*. These species represent both common vectors of disease and species that rarely encounter humans, exhibiting a range of host preferences and natural history. We found significant differences in microbial species diversity and composition by tick species and life stage. The microbiome of most species examined were dominated by a few primary endosymbionts. Across all species, the relative abundance of these endosymbionts increased with life stage while species richness and diversity decreased with development. Only one species, *I. angustus*, did not show the presence of a single dominant microbial species indicating the unique physiology of this species or its interaction with the surrounding environment. Tick species that specialize in a small number of host species or habitat ranges exhibited lower microbiome diversity, suggesting that exposure to environmental conditions or host blood meal diversity can affect the tick microbiome which in turn may affect pathogen transmission. These findings reveal important associations between ticks and their microbial community and improve our understanding of the function of non-pathogenic microbiomes in tick physiology and pathogen transmission.

## 1. Introduction 

Ticks are some of the most important vectors of diseases to humans and other animal hosts [1]. In addition to the pathogens they transmit, ticks can also harbor numerous symbiotic and commensal microbes such as bacteria, fungi, viruses, and protozoa [2,3,4]. These microbes, particularly the bacterial constituents, are increasingly recognized as important components of the tick microbiome that may interact with tick-borne pathogen transmission [5,6]. For instance, it has long been observed that a commensal bacteria in *Dermacentor andersoni* ticks limits the distribution of the pathogen that causes Rocky Mountain Spotted Fever (*Rickettsia rickettsii*) [7]. More recently, the transmission of the livestock pathogen, *Anaplasma marginale*, was inhibited by higher proportions and quantities of an endosymbiotic bacteria, *Rickettsia bellii* [8]. Overall tick microbial diversity has also been found to influence the colonization success of the Lyme disease pathogen, *Borrelia burgdorferi* [9]. In particular, an experimental study found that higher bacterial diversity affected the quality of the midgut lining and facilitated *B. burgdorferi* transmission into *Ixodes scapularis* [9]. 

Despite the growing appreciation that tick-associated microbiomes can be important to vector competency and pathogen transmission dynamics [8,9,10,11], the bacterial communities of many tick species, particularly those that are not common human disease vectors, have yet to be investigated. Comparisons of microbiome compositions and endosymbiont patterns between tick species may be a valuable path forward to better understand how tick microbiomes are shaped and perhaps how they influence vector competency [12]. Amplicon-based next-generation sequencing of the bacterial microbiome, hereafter referred to as the microbiome, is an effective and efficient method that allows for rapid characterization of the entire bacterial community in ticks [13,14]. Despite this recent technological advancement, there have been few studies that have examined how the tick microbiome changes through time or as the tick develops from one life stage to the next, but see [11,15]. Since hard ticks take a single blood meal during each of its post-egg life stages, comparative microbiome analyses of tick life stages could reveal how host blood meal or tick natural history affect the tick microbiome. Further, for public health reasons, tick microbiome studies have focused on generalist tick species that tend to transmit zoonotic diseases [8,9,10,15,16,17,18]. However, in the far-western United States, the co-occurrence of generalist ticks and several nest-dwelling, host specialist ticks provides an opportunity for comparative microbiome analyses of ticks with divergent life-history strategies [19,20,21]. 

In the western United States, there are numerous hard tick species (Family Ixodidae) with sympatric distributions. Many of these are important vectors for human diseases. *Ixodes pacificus* is endemic to the coastal and high elevation regions of western North America and is the main vector for *B. burgdorferi* [22,23]. Other tick species like *Dermacentor albipictus*, *Dermacentor occidentalis*, *Dermacentor variabilis*, *Ixodes angustus*, and *Haemaphysalis leporispalustris* are also present in this region [19,20,21,22,24]. While *D. occidentalis* and *D. variabilis* are vectors of human pathogens, such as Rocky Mountain Spotted Fever [25,26], tularemia [27], and the newly described Pacific Coast tick fever [28], *H. leporispalustris* and *D. albipictus* are host specialist ticks that do not frequently bite humans and are therefore not common zoonotic vectors [20,29]. In particular, *H. leporispalustris* is a nest-dwelling rabbit specialist and rarely quests in the open [30]. Meanwhile, the winter tick, *D. albipictus*, is a one-host tick that quests for a large ungulate host as a larva and then spends the next two life stages feeding and later breeding on the same individual host [29]. Due to this highly specialized host association, *D. albipictus* is not usually considered a zoonotic vector but recent work has shown that it is the infrequent vector of babesiosis in the western United States [31]. Although *I. angustus* can feed on a variety of rodent species and has been shown to be competent for transmitting *B. burgdorferi* [32] and *Anaplasma phagocytophilum* [33], it rarely bites humans and has a narrower habitat distribution than other *Ixodes* spp. [21].

In this study, we sought to investigate the species-specific microbiomes of six sympatric tick species that represent a diversity of natural histories and vary in their capacity to transmit zoonotic pathogens. We addressed how their microbiome communities change through ontogenic development and ask how life history (e.g., generalist vs. specialist) affects the diversity and composition of the tick microbiome. We focus on tick species endemic to north coastal California, a region with high diversity and sympatry of ixodid ticks to better understand the non-pathogenic microbial component of different species and factors responsible for structuring tick microbiomes more broadly. 

## 2. Materials and Methods

### 2.1. Sample Collection

Ticks were collected from Jack London State Park (38°21′24.1″ N 122°32′27.2″ W), China Camp State Park (38°00′09.7″ N 122°28′01.2″ W), the University of California Santa Cruz Forest Ecology Research Plot (FERP) (37°00′45.7″ N 122°04′25.1″ W), the Presidio Golden Gate National Recreation Area of San Francisco (37°47′55.7″ N 122°27′58.3″ W), and Rancho Murieta in Sacramento (38°30′06.5″ N 121°05′40.9″ W) in 2015. Most ticks were collected by dragging 1 m^2^ white flannel flags over the forest understory. *Dermacentor albipictus* nymphs and adults were collected off hosts (California mule deer and bighorn sheep) by the California Department of Public Health as this species spends the majority of its life cycle on ungulate hosts after the initial blood meal (Swei et al., 2018). Larval *I. angustus* were obtained from eggs hatched in the lab to engorged adult female ticks collected from small mammals at the FERP because this species is primarily nest-dwelling and difficult to collect using standard drag techniques. One *I. angustus* nymph was collected by drag sampling in the field. Immediately after collection, ticks were flash-frozen in liquid nitrogen and stored at −80 °C until laboratory identification of species, sex, and life stage. Once identified, ticks were stored at −20 °C until DNA extraction. 

### 2.2. DNA Extraction

To remove external environmental contaminants, ticks were surface sterilized using successive washes with 3% hydrogen peroxide, 70% ethanol, and ddH_2_O. Whole ticks were then ground using sterilized pestles, and DNA was extracted individually using the Qiagen DNeasy Blood and Tissue Kit (Qiagen, Inc., Valencia, CA, USA) as specified in the manufacturers’ instructions. Extracted DNA was stored at −20 °C until library preparation for sequencing. 

### 2.3. Sample Preparation

Separate 16S rRNA libraries were prepared for each tick sample following the guidelines in Klindworth et al., 2013 [14]. First, amplicon PCR was performed using primers from Klindworth et al., [14] and following the procedure in the Illumina 16S Metagenomic Sequencing library preparation manual (Illumina, Inc., San Diego, CA, USA). We targeted the 16S rRNA V3-V4 hypervariable region as sequencing this region enables identification of a broad range of bacteria that are relevant to ticks and their environment and is a common target in tick microbiome studies [10,13,14,34]. Amplicon PCRs were performed in triplicate for each sample, and the resulting product was pooled to reduce amplification bias. Samples were then purified via solid-phase reversible immobilization (SPRI) beads [13] or via gel extraction using the Accuprep PCR purification kit (Bioneer, Alameda, CA, USA) following the manufacturers’ instructions. Dual indices were then attached to the purified amplicons by PCR using primers from the Nextera XT v2 Index Kit set (Illumina, Inc., San Diego, CA, USA). Each sample was amplified in duplicate, pooled, and purified as above. Library concentrations were then quantified via qPCR using the primers and protocol provided in the KAPA Library Quantification kit (Kapa Biosciences, Woburn, MA, USA). Finally, all samples were diluted to a 2 nM concentration and pooled to form a multiplexed library. The combined library contained 2 negative controls originating from the DNA extraction step which were later used to identify and remove suspected contaminants. 

### 2.4. Library Sequencing

The final library was sequenced on an Illumina MiSeq using a MiSeq Reagent Kit v3 (600-cycle, 300 base pair, paired-end) (Illumina, Inc., San Diego, CA, USA). 

### 2.5. Sequence Processing

Microbiome sequencing processing was conducted using Quantitative Insights in Microbial Ecology (QIIME) [35] and R v3.4. Fastq sequence data were demultiplexed and quality-filtered (at Phred Q20). Paired-end reads were aligned, assigned to operational taxonomic units (OTUs) using 97% sequence similarity, and rarefied to 7000 reads per sample to correct for uneven sampling. Taxonomy was assigned using open-reference OTU picking and the NBCI database. Using the resulting OTU table, all OTUs present at less than 1% in all samples were pooled into a rare genera category, to minimize the impact of sequencing artifacts on diversity estimates [36]. Remaining suspected contaminants were then removed using the decontam package in R v 3.4, which identifies OTUs more abundant in negative controls than real samples [37].

### 2.6. Diversity Analysis

Microbiome richness, evenness, and Shannon’s diversity calculations were performed on the quality-filtered OTU table described above and were conducted at the genus level. These analyses were conducted using the vegan package in R. Statistical significance in diversity estimates between groups (species, life stage, and region) was determined using the Kruskal-Willis test as the data were not normally distributed. Microbiome profiles were also assessed for adult ticks by sex. 

### 2.7. Microbiome Composition Analysis

Microbiome community composition was compared amongst groups using the vegdist function in the vegan package. Two community dissimilarity metrics were used to capture different features of community composition—the Bray-Curtis dissimilarity index, which accounts for OTU abundance, and the Jaccard index which accounts for only OTU presence/absence. Community dissimilarity between groups was then compared via PerMANOVA using 999 permutations of the distance values as a comparison. 

### 2.8. Core Microbiome Analysis

We sought to identify the microbes with the strongest association with each tick species, as these microbes likely serve an important ecological and functional role within the tick. These “core microbiome” members were selected based on criteria adapted from [38]. Specifically, the core microbiota for a given tick species were those: (1) present in ticks from all locations sampled, (2) present in >50% of all individuals sampled, and (3) present at >5% relative abundance as determined by the percentage of sequence reads attributed to that microbe. 

### 2.9. Predicted Microbiome Function Analysis

We estimated the functional role of each microbial community using PICRUSt v1.1 [39], a computational approach that uses 16S rRNA sequencing information and a reference database to infer functional gene content. Using PICRUSt and the KEGG orthology database [40], we grouped gene content predictions to the default hierarchical level (level 3). We then compared predicted gene family content across treatments via ANOVA and applied an FDR multiple testing correction. The accuracy of the PICRUSt estimates was assessed by calculating the weighted Nearest Sequenced Taxon Index (NSTI) which measures the availability of nearby genome representatives for the given OTUs. 

## 3. Results

### 3.1. Sample Numbers

We collected a total of 143 ticks from six species for microbiome analysis (Supplementary Table S1). Three of the six species had ticks from each post-egg life stage (*D. albipictus*, *D. occidentalis*, and *I. pacificus*) while we were only able to collect adult *D. variabilis*, larval *H. leporispalustris*, and larval and nymphal *I. angustus*. Sequencing these samples yielded 7,907,960 reads passing quality filter. All raw sequence files are accessioned at Sequence Read Archive under BioProject ID PRJNA574713. After removing suspected contaminants and pooling rare genera [11], we found a total of 59 OTUs across all sample types.

### 3.2. Tick Species Microbiome Differences

**Figure 1:**
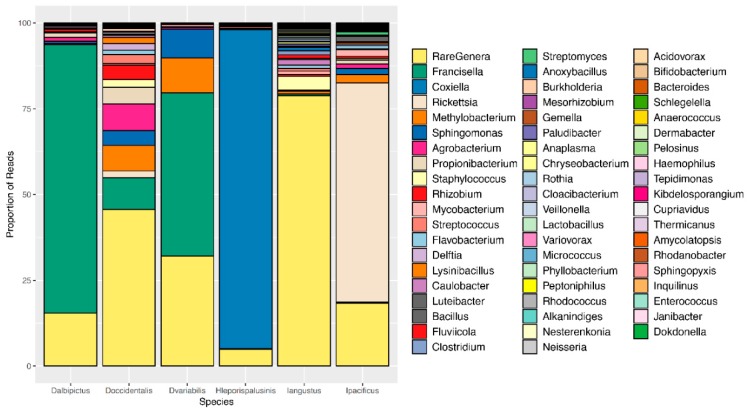
Microbiome composition by tick species. A representative, or averaged, microbiome is shown for each tick species with all life stages included. Colors represent OTUs at the genus level, and bar heights correspond to OTU relative abundance as determined by the percentage of sequence reads.

**Figure 2:**
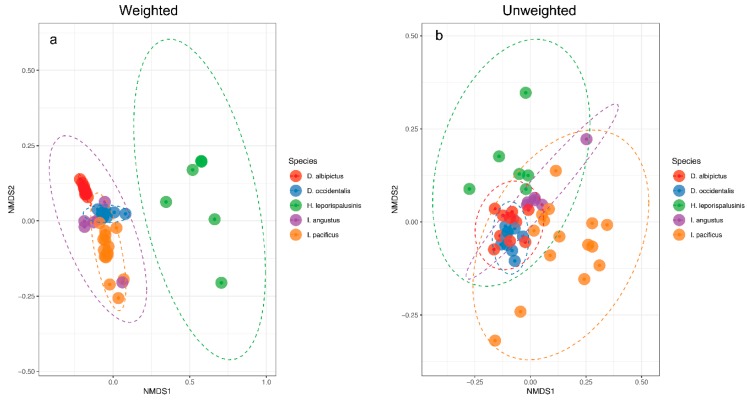
Microbiome representation of larvae by species. (**a**) Weighted and (**b**) Unweighted non-metric multidimensional scaling (NMDS) by species at the larval life stage. Ellipses represent a 95% confidence interval around the centroid of each group.

We observed pronounced differences in the microbial communities of sympatric tick species (Figure 1). Specifically, microbiome composition differed significantly by both the Jaccard and Bray-Curtis dissimilarity metrics (*df* = 5, *F* = 4.82, *p* = 0.001, *df* = 5, *F* = 6.41, *p* = 0.001, Figure 2, Supplementary Figures S1 and S2). Microbial richness also differed significantly between tick species at all life stages, and microbial diversity differed significantly between species at the larval and adult life stages (*p* < 0.05 for all, Supplementary Tables S2 and S3). 

**Figure 3:**
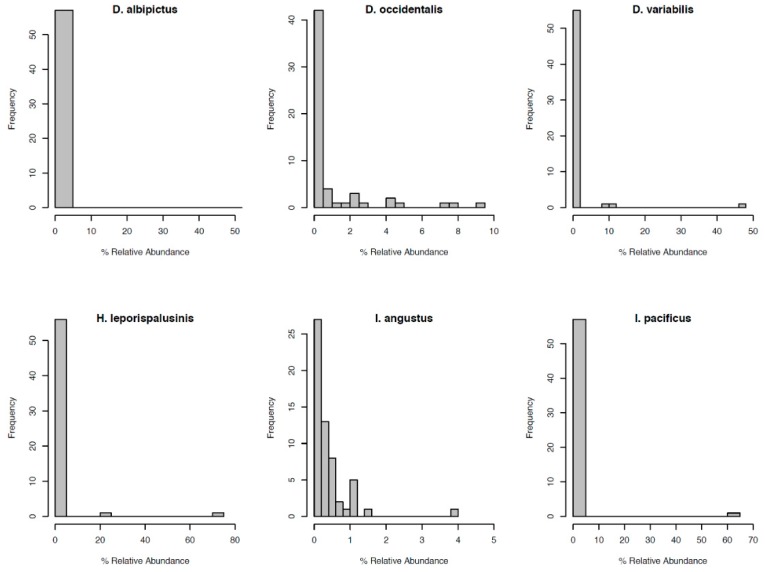
Distribution of OTU relative abundance for each species’ microbiome. The x-axis denotes the percentage of sequence reads attributed to a given OTU. The y-axis denotes the number of OTUs occurring at a given relative abundance.

Most notably, the core microbiota—the OTUs most frequently present at the greatest abundance within the microbiome—varied between tick species (Table 1). *Dermacentor variabilis* and *D. occidentalis* were both dominated by *Francisella, Sphinogomonas*, and *Methylobacterium*, while *D. albipictus* was dominated only by *Francisella*. The dominant members of *H*. *leporispalustris* and *I. pacificus* were *Coxiella* and *Rickettsia*, respectively, while *I. angustus* had no discernible core microbiome (Table 1). Sequences obtained from core microbiota OTU were aligned to the Genbank nucleotide sequence database using NCBI Blast and the top matches are presented in Supplementary Table S4. In addition to identity, the relative abundance of these core microbes varied significantly by species (ANOVA *F* = 25.62, *df* = 5, *p* < 0.001, Supplementary Figure S3). All tick microbiomes displayed highly right-skewed abundance distributions (Figure 3), indicating that for all tick species, the microbiomes contained many rare OTUs but were dominated by a few highly abundant taxa. The relative abundance of rare OTUs that never comprise more than 1% of the sequence reads in any sample, differed significantly between tick species (ANOVA *F* = 28.31, *df* = 5, *p* < 0.001, Figure 1 and Supplementary Figure S4).

When comparing the functional gene content of tick microbiomes, none of the 328 predicted gene pathways differed significantly by species at the larval life stage. However, 14 out of 328 gene pathways varied significantly when comparing only larval *I. pacificus* and *I. angustus*, two genetically similar species with diverging life-history strategies (Supplementary Table S5). At the nymphal life stage, 255 out of 328 gene pathways differed significantly by species (Supplementary Table S5). The average weighted Nearest Sequenced Taxon Index (NSTI) for our samples was 0.035 (sd = 0.007) indicating that our samples were highly tractable for metagenome prediction [39].

### 3.3. Ontogenic Microbiome Changes

**Figure 4:**
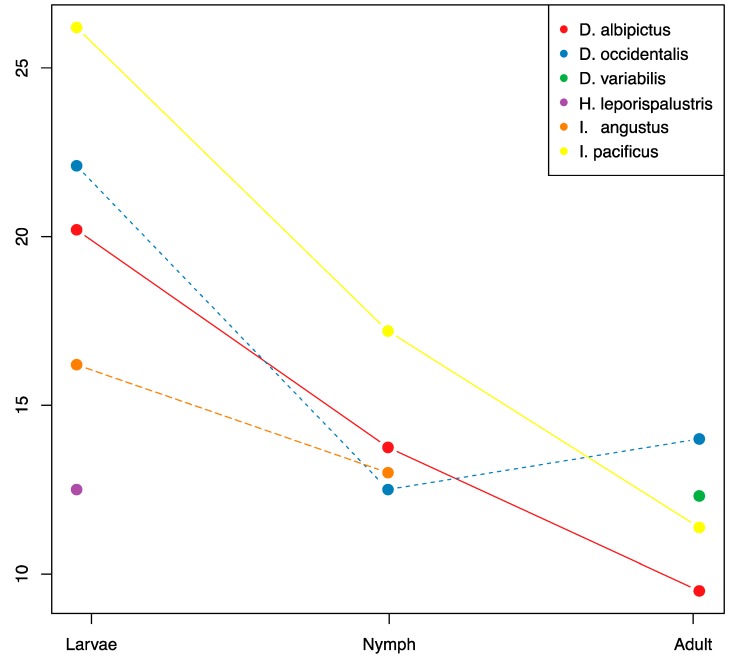
OTU richness through ontogenic development. Mean OTU richness is plotted through life stages for each species with sufficient coverage. Sample numbers for each treatment are listed in Supplementary Table S1.

There were significant reductions in microbiome species richness and diversity through development for all tick species with sufficient sample coverage (*D. albipictus*, *D. occidentalis*, and *I. pacificus*, Figure 4, Supplementary Tables S1 and S5). On average the adult stages had approximately 50% of the microbiome richness of larvae, and nymphs exhibited intermediate richness. We found a consistent pattern of OTU loss through tick development with larvae exhibiting the highest levels of species richness and adults exhibiting the lowest levels. Only one species, *D. occidentalis*, exhibited a slight increase in species richness from one developmental life stage to the next (nymph to adult). When analyzing all species together, we detected significant differences in microbiome composition by life stage using the Jaccard, presence/absence-based dissimilarity metric (*df* = 2, *F* = 5.67, *p* = 0.007), but not for the Bray-Curtis, abundance-based dissimilarity metric (*df* = 2, *F* = 2.74, *p* = 0.077). 

Microbiome differences of adult ticks by species and sex found that for many species, the profiles were similar with the exception of *D. variabilis* for which *Francisella* was present at a higher relative abundance in females compared to males (Supplemental Figure S5).

### 3.4. Regional Microbiome Differences

We compared larval *I. pacificus*, *D. albipictus*, and *D. occidentalis* collected from two sites located approximately 40 miles apart to examine the effects of geographic variation on tick microbiomes. We detected no significant differences in microbial richness, diversity, or composition between ticks collected from these two regions (Supplementary Tables S6 and S7, Supplementary Figure S6). Further, none of the 328 predicted gene pathways differed significantly by region. 

## 4. Discussion

We conducted a multi-species comparative microbiome analysis of ixodid tick species and found that distinct microbial compositions, diversity, and core microbes characterized these sympatric tick species. The species examined ranged from highly generalist species like the Lyme disease vector, *I. pacificus*, which feeds on a wide diversity of hosts [19] to extreme host specialists like *D. albipictus* which feeds on a single host over the course of its three life stages [29]. Consistent with other studies of tick microbiomes [8,10,16], our analysis found that hard tick microbiomes are heavily dominated by a few core species, likely endosymbionts [41]. These tick-symbiont relationships appear to be relatively stable [11] and are important for tick physiology and may interact with pathogens as well [12]. *Ixodes pacificus* is uniformly associated with an endosymbiotic *Rickettsia* [41], whereas we found that *D. occidentalis and D. variabilis* are associated with *Francisella*, *Sphinogomonas*, and *Methylobacterium* (Table 1), consistent with other studies [3,42]. While the function of endosymbionts in tick physiology is not entirely known, their generally ubiquitous occurrence in hard ticks suggests they serve an essential function in these hematophagous tick parasites, perhaps in the form of nutritional supplementation of folic acid and other nutrients lacking in blood [12,41,43]. The one striking exception to this pattern was *I. angustus* which did not have a core microbiome, defined as having a microbial component present in a majority of samples and being present at greater than 5% relative abundance [38]. Although our sample size for *I. angustus* was small, we only found a single *Rickettsia* read among all the samples, demonstrating that a previously reported endosymbiont, *Candidatus* Rickettsia angustus [33], was not found in our samples and may not be essential. In contrast, the most dominant component of *I. angustus* microbiome was the “Rare genera” category (Supplementary Figure S4) indicating that most of the microbial species detected in the tick were present at less than 1% relative abundance of sequence reads. These results suggest that environmental or physiological factors that shape the tick microbiome are distinct in *I. angustus* and warrant further inquiry and investigation into the ecological and physiological factors that permit them to not require harboring a dominant endosymbiont. Experimental studies and habitat suitability models reveal that *I. angustus* is a nest dwelling specialist [44] and highly associated with coastal redwood forests in the northwestern US [21]. The unique environmental conditions or host associations in this habitat may enable *I. angustus* to survive and develop without an endosymbiont, though in some locations an endosymbiont has been reported [33], but the mechanisms driving this are unknown and require further study.

The relationship between tick species and their endosymbionts is likely a deep evolutionary relationship. *Ixodes* species such as *I. scapularis* and *I. ricinus* are also frequently associated with *Rickettsia* endosymbionts [9,16,17,45], although perhaps not to the same degree as *I. pacificus*. A comparison of microbiomes from different regions in the eastern United States found that while northern *I. scapularis* populations harbored high relative abundances of a *Rickettsia* symbiont, southern populations lacked *Rickettsia* and instead had high abundances of an Enterobacteriaceae [16]. Both ubiquitous and high relative abundance of *Rickettsia* endosymbionts in *I. pacificus* [46] implies an especially important relationship between endosymbiont and tick or a particularly strong ecological pressure driving the dominance of *Rickettsia* in the tick microbiome. Likewise, other studies of *Dermacentor* ticks show frequent association with *Francisella* endosymbionts [3,8,47].

While the core microbiota varied between tick species, general patterns did emerge from our species and life stage comparison of tick microbial diversity. We find that across the tick species examined, microbial species richness tended to be highest in the larval stage and decreased with each subsequent life stage until the adult microbiome becomes almost entirely composed of the dominant endosymbiont(s), a finding that is consistent with studies of other tick species [48]. This pattern is true both for generalist, multi-host ticks like *I. pacificus* as well as for the one-host specialist, *D. albipictus*. It should be noted that nymph and adult *D. albipictus* samples were partially engorged due to the necessity of collecting them from their blood meal host and it is not clear if the host blood would tend to increase or decrease the OTU richness signature, however, host blood is generally low in bacterial richness [49]. Due to sampling limitations, we did not have nymphal or adult *H. leporispalustris* samples, therefore we were not able to assess patterns in these life stages in this rabbit specialist species. It is unclear if the loss of microbial species richness is driven by competitive interactions between components of the microbiome or perhaps reflects the gradual loss of transient, unstable microbes that are more commonly associated with the larval stage [11,50]. It has also been suggested that the tick host itself is filtering non-essential or harmful microbes after initial, transient colonization from environmental sources [11]. Our results suggest that this pattern may be common among many hard tick species and could indicate a conserved microbial successional pattern in hard ticks. Further studies should seek to better understand how and why microbial diversity is lost through the tick’s ontogenic development.

Although microbial richness was highest at the larval stage, the predicted gene function did not differ at all between species at this life stage. At the nymphal stage, however, there were large numbers of differences in predicted gene function despite a loss of microbial species richness. Our finding that predicted gene function was conserved across all species at the larval stage but started to diverge at the nymphal stage suggests that tick age or blood meal host differences may be driving these functional differences. Perhaps species-specific differences in natural history like host blood meal associations or questing behavior play a role in driving these functional differences via changes in microbiome composition [10]. While it is still not clear how comprehensive these gene prediction algorithms are, these results suggest that there is still much to learn about what factors influence the composition and function of the tick microbiome and how tick microbial activity may affect tick fitness or pathogen transmission. 

In this study, we collected as many life stages from the field as possible from a range of sympatric ixodid tick species. Some of these species are generalist ticks and important zoonotic vectors such as *I. pacificus* and *D. variabilis*, but others have highly specialized host preferences like *D. albipictus* and *H. leporispalustris*, and rarely transmit pathogens to humans. Generalist ticks can feed on numerous species of hosts such as rodents, birds, and lizards [19,33] while specialist ticks feed predominantly on a single or limited range of species such as rabbits in the case of *H. leporispalustris* or large ungulates in the case of *D. albipictus*. We found that *H. leporispalustris* had significantly lower microbiome species richness and diversity compared to the other species at the larval stage. In addition, the large ungulate one-host tick, *D. albipictus* had lower microbiome species diversity (Table S3) and distinct microbial communities (Supplementary Figure S6) compared to the other *Dermacentor* spp., especially at the larval and adult stages. While the mechanisms behind these patterns are still unclear, these results suggest that a broader host range may contribute to greater microbiome diversity on a population level. Given that zoonotic vectors tend to be generalist species, higher microbial diversity may facilitate pathogen transmission, similar to what was found in *I. scapularis* [9]. 

The differences that we document are highly structured by tick species. We did not detect significant differences in microbial diversity, composition or function within a species between two regions where samples were collected. However, the distance between these two collection sites may not be far enough or sufficiently ecologically distinct to reflect potential microbiome differences. Expanded regional studies would be valuable to help tease apart inherent differences in tick microbiome composition versus factors that may be shaped by abiotic or biotic factors. 

Next-generation sequencing-based microbiome studies are improving our understanding of the relationship between tick microbiome composition, endosymbiont interactions and vector competency. These types of studies can help ecologists better understand microbial community ecology and also may also provide key insights into vector control to mitigate the emergence of vector-borne diseases, of which the majority are tick-borne diseases [51]. Our comparative tick microbiome study found differences in tick core microbiota but also common diversity patterns which may provide valuable insights into how ticks acquire and lose their microbiota, the function of endosymbionts, and how we can harness these relationships to control vector-borne disease transmission. We are still in the early stages of probing and understanding the drivers of and impacts of the tick microbiome but comparative studies like ours are a first step to developing testable hypotheses to better understand these relationships in the hope of being able to better mitigate tick-borne disease transmission. 

## Figures and Tables

**Table 1:** The core microbiome members for each tick species.

Tick Species	Core Microbiome
*D. albipictus*	*Francisella*
*D. occidentalis*	*Francisella, Sphinogomonas, Methylobacterium*
*D. variabilis*	*Francisella, Sphinogomonas, Methylobacterium*
*H. leporispalustris*	*Coxiella*
*I. angustus*	None
*I. pacificus*	*Rickettsia*

## Supplementary Materials

The following are available online at https://www.mdpi.com/2075-4450/10/10/353/s1, Figure S1: Microbiome representation of nymphs by species, Figure S2: Microbiome representation of adults by species, Figure S3: Relative abundance of core microbiota by tick species, Figure S4: Relative abundance of rare genera (See Methods: Sequence processing) by tick species, Figure S5: Microbiome composition by tick species and adult sex, Figure S6: Microbiome representation of larval *D. albipictus*, *D. occidentalis*, and *I. pacificus* by region (China Camp State Park in Marin, CA or Jack London State Park in Sonoma, CA., Table S1: Sample Size for Treatment Groups, Table S2: Species Richness by Species & Life Stage, Table S3: Species Diversity by Species & Life Stage, Table S4: Top sequence BLAST results from OTUs identified as core microbiota, Table S5: Variation in functional gene content by sample type, Table S6: OTU Richness by Location, Table S7: OTU Diversity by Location.
