# Supplementary material for: Comparative Microbiome Profiles of Sympatric Tick Species from the Far-Western United States
Source: Insects. 2019 Oct 18;10(10):353. doi: 10.3390/insects10100353 (PMC6836157; doi:10.3390/insects10100353)
Supplement: Supplementary file 1 [file insects-10-00353-s001.zip › insects-597620-SI.docx]

Supplementary Material

**Supplementary Table S1. Sample Size for Treatment Groups.** Values listed denote the number of ticks collected and sequenced for each life stage and species.

|  | **All Life Stages** | **Larvae** | **Nymph** | **Adult** |
| --- | --- | --- | --- | --- |
| *D.albipictus* | 34 | 10 | 8 | 16 |
| *D.occidentalis* | 31 | 10 | 6 | 15 |
| *D.variabilis* | 13 | 0 | 0 | 13 |
| *H.leporispalustris* | 6 | 6 | 0 | 0 |
| *I.angustus* | 6 | 5 | 1 | 0 |
| *I.pacificus* | 49 | 15 | 10 | 24 |
| All Species | 143 | 46 | 27 | 70 |

**Supplementary Table S2. Species Richness by Species & Life Stage.** Values listed denote the mean and 1 standard deviation. Statistical significance is determined via Kruskal-Wallis test, with (*) denoting a p-value < 0.05, (**) for a p-value of < 0.01, and (†) for groups in which there is only one treatment.

|  | **All Life Stages*** | **Larvae**** | **Nymph*** | **Adult**** |
| --- | --- | --- | --- | --- |
| *D.albipictus*** | 13.65 ± 5.24 | 20.2 ± 2.70 | 13.75 ± 2.76 | 9.50 ± 2.34 |
| *D.occidentalis*** | 16.32 ± 4.81 | 22.1 ± 3.31 | 12.50 ± 2.07 | 14.0 ± 2.27 |
| *D.variabilis* † | 12.31 ± 2.36 | NA | NA | 12.31 ± 2.36 |
| *H.leporispalustris* † | 13.13 ± 2.75 | 12.5 ± 2.88 | NA | NA |
| *I.angustus* | 15.67 ± 2.58 | 16.2 ± 2.49 | 13.00 | NA |
| *I.pacificus*** | 17.10 ± 7.19 | 26.2 ± 3.84 | 17.20 ± 2.66 | 11.38 ± 2.84 |
| All Species** | 15.30 ± 5.78 | 21.3 ± 5.60 | 14.81 ± 3.05 | 11.66 ± 2.94 |

**Supplementary Table S3.** **Species Diversity by Species & Life Stage.** Values listed denote the mean and 1 standard deviation. Statistical significance is determined via Kruskal-Wallis test, with (*) denoting a p-value < 0.05, (**) for a p-value of < 0.01, and (†) for groups in which there is only one treatment.

|  | **All Life Stages**** | **Larvae**** | **Nymph** | **Adult**** |
| --- | --- | --- | --- | --- |
| *D.albipictus** | 0.64 ± 0.36 | 0.85 ± 0.23 | 0.73 ± 0.44 | 0.46 ± 0.30 |
| *D.occidentalis*** | 1.50 ± 0.51 | 2.10 ± 0.15 | 1.38 ± 0.54 | 1.14 ± 0.18 |
| *D.variabilis* † | 0.92 ± 0.38 | NA | NA | 0.92 ± 0.38 |
| *H.leporispalustris* † | 0.31 ± 0.19 | 0.31 ± 0.18 | NA | NA |
| *I.angustus* | 0.94 ± 0.20 | 0.99 ± 0.18 | 0.70 | NA |
| *I.pacificus *** | 1.08 ± 0.69 | 1.90 ± 0.34 | 1.03 ± 0.51 | 0.58 ± 0.33 |
| All Species** | 1.01 ± 0.62 | 1.41 ± 0.70 | 1.00 ± 0.53 | 0.74 ± 0.40 |

**Supplementary Table S4.** **Top sequence BLAST results from OTUs identified as core microbiota.** Sequences are based on the 16S rRNA V3-V4 hypervariable region.

| Tick species | Core microbiota genus | Description | Percent Identity | Accession |
| --- | --- | --- | --- | --- |
| *Dermacentor variabilis, Dermacentor occidentalis, Dermacentor albipictus* | *Francisella* | Uncultured *Francisella* sp. clone 60-2 | 99.36% | MH595917 |
|  |  | *Francisella* endosymbiont of *Dermacentor variabilis* isolate Dv0866_CA | 99.36% | MG834501 |
| *Dermacentor variabilis, Dermacentor occidentalis* | *Methylobacterium* | *Methylobacterium* sp. WL1 | 99.77% | CP042823 |
|  |  | *Methylobacterium oryzae* strain MPA1 | 99.77% | MN174141 |
|  |  | *Methylobacterium radiotolerans* CP39.2 | 99.77% | MK968428 |
|  |  | *Methylobacterium brachiatum* G93 | 99.77% | MH930110 |
|  |  | *Methylobacterium phyllostachyos* 0B28 | 99.77% | MH929655 |
|  |  | *Methylobacterium tardum* R.M3S1 | 99.77% | MH298496 |
| *H. leporispalustris* | *Coxiella* | *Coxiella* endosymbiont of *Ornithodoros sonrai* isolate Oson2 | 96.77% | KP994798 |
|  |  | Coxiella endosymbiont of *Haemaphysalis punctata* isolate Haepun3 | 96.56% | MH645196 |
|  |  | *Coxiella*-like endosymbiont of *Rhipicephalus bursa* clone P14 | 96.36% | MH645195 |
|  |  | *Coxiella burnetii* strain Heizberg | 95.91% | CP014561 |
| *I. pacificus* | *Rickettsia* | *Rickettsia monacensis* 1187_ISE6 | 99.77% | LC388771 |
|  |  | *Rickettsia japonica* strain Shandong J75 | 99.77% | MF496161 |
|  |  | *Rickettsia buchneri* strain ISO7 | 99.77% | NR_134842 |
|  |  | Rickettsia endosymbiont of *Ixodes pacificus* isolate CP-1 | 99.77% | KP276591 |
| *Dermacentor variabilis, Dermacentor occidentalis* | *Sphingomonas* | *Sphingomonas* sp. strain Os1-19 | 99.77% | KY908295 |

**Supplementary Table S5.** Variation in functional gene content by sample type. This table lists the predicted functional gene pathways, collapsed at the default, 3^rd^ hierarchical level. An ‘X’ indicates that this pathway was differentially expressed across treatment types at P< 0.05 after FDR correction for multiple testing.

| Gene Pathway | *I. pacificus* vs *I. angustus* (larvae only) | All species (nymphs only) |
| --- | --- | --- |
| 1,1,1-Trichloro-2,2-bis(4-chlorophenyl)ethane (DDT) degradation |  |  |
| ABC transporters |  | X |
| Adherens junction |  | X |
| Adipocytokine signaling pathway |  | X |
| African trypanosomiasis |  |  |
| Alanine, aspartate and glutamate metabolism |  | X |
| Aldosterone-regulated sodium reabsorption |  | X |
| Alzheimer's disease |  | X |
| Amino acid metabolism |  | X |
| Amino acid related enzymes |  | X |
| Amino sugar and nucleotide sugar metabolism |  | X |
| Aminoacyl-tRNA biosynthesis | X |  |
| Aminobenzoate degradation |  | X |
| Amoebiasis |  | X |
| Amyotrophic lateral sclerosis (ALS) |  | X |
| Antigen processing and presentation |  | X |
| Apoptosis |  | X |
| Arachidonic acid metabolism |  |  |
| Arginine and proline metabolism |  | X |
| Arrhythmogenic right ventricular cardiomyopathy (ARVC) |  | X |
| Ascorbate and aldarate metabolism | X | X |
| Atrazine degradation |  | X |
| Bacterial chemotaxis | X | X |
| Bacterial invasion of epithelial cells |  | X |
| Bacterial motility proteins |  | X |
| Bacterial secretion system |  | X |
| Bacterial toxins |  | X |
| Basal transcription factors |  | X |
| Base excision repair |  | X |
| Benzoate degradation |  | X |
| Betalain biosynthesis |  | X |
| Bile secretion |  |  |
| Biosynthesis and biodegradation of secondary metabolites |  | X |
| Biosynthesis of 12-, 14- and 16-membered macrolides |  | X |
| Biosynthesis of ansamycins |  |  |
| Biosynthesis of siderophore group nonribosomal peptides |  |  |
| Biosynthesis of type II polyketide backbone |  | X |
| Biosynthesis of type II polyketide products |  | X |
| Biosynthesis of unsaturated fatty acids |  | X |
| Biosynthesis of vancomycin group antibiotics |  | X |
| Biotin metabolism |  | X |
| Bisphenol degradation |  | X |
| Bladder cancer |  | X |
| Butanoate metabolism |  | X |
| Butirosin and neomycin biosynthesis |  |  |
| C5-Branched dibasic acid metabolism |  | X |
| CAM ligands |  |  |
| Caffeine metabolism |  | X |
| Calcium signaling pathway |  | X |
| Caprolactam degradation |  | X |
| Carbohydrate digestion and absorption |  | X |
| Carbohydrate metabolism |  | X |
| Carbon fixation in photosynthetic organisms | X | X |
| Carbon fixation pathways in prokaryotes |  | X |
| Cardiac muscle contraction |  |  |
| Carotenoid biosynthesis |  | X |
| Cell cycle |  |  |
| Cell cycle - Caulobacter | X | X |
| Cell cycle - yeast | X | X |
| Cell division |  | X |
| Cell motility and secretion |  | X |
| Cellular antigens |  | X |
| Chagas disease (American trypanosomiasis) |  | X |
| Chaperones and folding catalysts |  | X |
| Chloroalkane and chloroalkene degradation |  | X |
| Chlorocyclohexane and chlorobenzene degradation |  |  |
| Cholinergic synapse |  |  |
| Chromosome |  | X |
| Chronic myeloid leukemia |  | X |
| Circadian rhythm - plant |  |  |
| Citrate cycle (TCA cycle) |  | X |
| Clavulanic acid biosynthesis |  |  |
| Colorectal cancer |  | X |
| Complement and coagulation cascades |  | X |
| Cyanoamino acid metabolism |  | X |
| Cysteine and methionine metabolism |  |  |
| Cytochrome P450 |  |  |
| Cytokine receptors |  | X |
| Cytokine-cytokine receptor interaction |  |  |
| Cytoskeleton proteins |  | X |
| Cytosolic DNA-sensing pathway |  | X |
| D-Alanine metabolism |  | X |
| D-Arginine and D-ornithine metabolism |  | X |
| D-Glutamine and D-glutamate metabolism |  | X |
| DNA repair and recombination proteins |  | X |
| DNA replication |  |  |
| DNA replication proteins |  | X |
| Dilated cardiomyopathy (DCM) |  | X |
| Dioxin degradation |  | X |
| Drug metabolism - cytochrome P450 |  |  |
| Drug metabolism - other enzymes |  | X |
| ECM-receptor interaction |  |  |
| Electron transfer carriers |  | X |
| Endocrine and other factor-regulated calcium reabsorption |  | X |
| Endocytosis |  | X |
| Energy metabolism |  | X |
| Epithelial cell signaling in Helicobacter pylori infection |  | X |
| ErbB signaling pathway |  | X |
| Ether lipid metabolism | X | X |
| Ethylbenzene degradation |  | X |
| Fat digestion and absorption |  |  |
| Fatty acid biosynthesis |  | X |
| Fatty acid elongation in mitochondria |  |  |
| Fatty acid metabolism |  | X |
| Fc epsilon RI signaling pathway |  | X |
| Fc gamma R-mediated phagocytosis |  | X |
| Flagellar assembly |  | X |
| Flavone and flavonol biosynthesis |  | X |
| Flavonoid biosynthesis |  |  |
| Fluorobenzoate degradation |  | X |
| Focal adhesion |  | X |
| Folate biosynthesis |  | X |
| Fructose and mannose metabolism |  |  |
| Function unknown |  |  |
| G protein-coupled receptors |  | X |
| GTP-binding proteins |  |  |
| Galactose metabolism |  | X |
| Gastric acid secretion |  | X |
| General function prediction only | X | X |
| Geraniol degradation |  |  |
| Germination |  | X |
| Glioma |  | X |
| Glutamatergic synapse |  |  |
| Glutathione metabolism | X | X |
| Glycan bindng proteins |  | X |
| Glycan biosynthesis and metabolism |  | X |
| Glycerolipid metabolism |  | X |
| Glycerophospholipid metabolism |  | X |
| Glycine, serine and threonine metabolism |  |  |
| Glycolysis / Gluconeogenesis |  | X |
| Glycosaminoglycan biosynthesis - chondroitin sulfate |  | X |
| Glycosaminoglycan degradation |  | X |
| Glycosphingolipid biosynthesis - ganglio series |  |  |
| Glycosphingolipid biosynthesis - globo series |  |  |
| Glycosphingolipid biosynthesis - lacto and neolacto series |  | X |
| Glycosylphosphatidylinositol(GPI)-anchor biosynthesis |  | X |
| Glycosyltransferases |  | X |
| Glyoxylate and dicarboxylate metabolism |  |  |
| GnRH signaling pathway |  |  |
| Hedgehog signaling pathway |  | X |
| Hematopoietic cell lineage |  | X |
| Hepatitis C |  | X |
| Histidine metabolism | X | X |
| Homologous recombination |  |  |
| Huntington's disease |  | X |
| Hypertrophic cardiomyopathy (HCM) |  | X |
| Indole alkaloid biosynthesis |  | X |
| Influenza A |  | X |
| Inorganic ion transport and metabolism |  | X |
| Inositol phosphate metabolism |  | X |
| Insulin signaling pathway |  |  |
| Ion channels |  | X |
| Isoflavonoid biosynthesis |  |  |
| Isoquinoline alkaloid biosynthesis |  |  |
| Leishmaniasis |  | X |
| Leukocyte transendothelial migration |  | X |
| Limonene and pinene degradation |  | X |
| Linoleic acid metabolism |  | X |
| Lipid biosynthesis proteins |  | X |
| Lipid metabolism | X | X |
| Lipoic acid metabolism |  | X |
| Lipopolysaccharide biosynthesis |  |  |
| Lipopolysaccharide biosynthesis proteins |  |  |
| Long-term depression |  | X |
| Long-term potentiation |  | X |
| Lysine biosynthesis |  | X |
| Lysine degradation |  |  |
| Lysosome |  | X |
| MAPK signaling pathway |  |  |
| MAPK signaling pathway - yeast |  | X |
| Measles |  |  |
| Meiosis - yeast |  | X |
| Melanogenesis |  | X |
| Membrane and intracellular structural molecules |  | X |
| Metabolism of cofactors and vitamins |  | X |
| Metabolism of xenobiotics by cytochrome P450 | X | X |
| Methane metabolism |  | X |
| Mineral absorption |  | X |
| Mismatch repair |  | X |
| N-Glycan biosynthesis |  | X |
| NOD-like receptor signaling pathway |  |  |
| Naphthalene degradation |  |  |
| Neuroactive ligand-receptor interaction |  | X |
| Neurotrophin signaling pathway |  | X |
| Nicotinate and nicotinamide metabolism |  | X |
| Nitrogen metabolism |  | X |
| Nitrotoluene degradation |  |  |
| Non-homologous end-joining |  | X |
| Notch signaling pathway |  | X |
| Novobiocin biosynthesis |  | X |
| Nucleotide excision repair |  |  |
| Nucleotide metabolism |  | X |
| Olfactory transduction |  |  |
| One carbon pool by folate |  | X |
| Oocyte meiosis |  | X |
| Other glycan degradation |  | X |
| Other ion-coupled transporters |  |  |
| Other transporters |  | X |
| Other types of O-glycan biosynthesis |  | X |
| Others |  | X |
| Oxidative phosphorylation |  |  |
| PPAR signaling pathway |  |  |
| Pancreatic cancer |  | X |
| Pancreatic secretion |  | X |
| Pantothenate and CoA biosynthesis |  |  |
| Parkinson's disease |  | X |
| Pathogenic Escherichia coli infection |  | X |
| Pathways in cancer |  | X |
| Penicillin and cephalosporin biosynthesis |  | X |
| Pentose and glucuronate interconversions |  | X |
| Pentose phosphate pathway |  | X |
| Peptidases |  | X |
| Peptidoglycan biosynthesis |  | X |
| Peroxisome |  |  |
| Pertussis |  | X |
| Phagosome |  | X |
| Phenylalanine metabolism |  | X |
| Phenylalanine, tyrosine and tryptophan biosynthesis |  | X |
| Phenylpropanoid biosynthesis |  | X |
| Phosphatidylinositol signaling system |  | X |
| Phosphonate and phosphinate metabolism |  | X |
| Phosphotransferase system (PTS) |  |  |
| Photosynthesis |  | X |
| Photosynthesis - antenna proteins |  |  |
| Photosynthesis proteins |  |  |
| Phototransduction |  | X |
| Phototransduction - fly |  | X |
| Plant-pathogen interaction |  | X |
| Polycyclic aromatic hydrocarbon degradation |  | X |
| Polyketide sugar unit biosynthesis |  | X |
| Pores ion channels |  | X |
| Porphyrin and chlorophyll metabolism |  | X |
| Prenyltransferases |  | X |
| Primary bile acid biosynthesis |  | X |
| Primary immunodeficiency |  | X |
| Prion diseases |  | X |
| Progesterone-mediated oocyte maturation |  | X |
| Propanoate metabolism |  | X |
| Prostate cancer |  | X |
| Proteasome |  | X |
| Protein digestion and absorption |  | X |
| Protein export |  | X |
| Protein folding and associated processing |  | X |
| Protein kinases |  | X |
| Protein processing in endoplasmic reticulum |  | X |
| Proximal tubule bicarbonate reclamation |  | X |
| Purine metabolism |  | X |
| Pyrimidine metabolism |  | X |
| Pyruvate metabolism |  | X |
| RIG-I-like receptor signaling pathway |  | X |
| RNA degradation |  | X |
| RNA polymerase |  |  |
| RNA transport |  | X |
| Regulation of actin cytoskeleton |  |  |
| Renal cell carcinoma |  | X |
| Renin-angiotensin system |  | X |
| Replication, recombination and repair proteins |  | X |
| Restriction enzyme |  |  |
| Retinol metabolism |  | X |
| Rheumatoid arthritis |  | X |
| Riboflavin metabolism |  | X |
| Ribosome |  | X |
| Ribosome Biogenesis |  |  |
| Ribosome biogenesis in eukaryotes | X | X |
| Salivary secretion |  | X |
| Secondary bile acid biosynthesis |  | X |
| Secretion system |  |  |
| Selenocompound metabolism |  | X |
| Sesquiterpenoid biosynthesis |  | X |
| Shigellosis |  | X |
| Signal transduction mechanisms |  | X |
| Small cell lung cancer |  |  |
| Sphingolipid metabolism | X | X |
| Spliceosome |  | X |
| Sporulation |  | X |
| Staphylococcus aureus infection |  | X |
| Starch and sucrose metabolism |  | X |
| Steroid biosynthesis |  | X |
| Steroid hormone biosynthesis |  | X |
| Stilbenoid, diarylheptanoid and gingerol biosynthesis |  | X |
| Streptomycin biosynthesis |  | X |
| Styrene degradation |  | X |
| Sulfur metabolism |  | X |
| Sulfur relay system |  |  |
| Synthesis and degradation of ketone bodies |  |  |
| Systemic lupus erythematosus |  | X |
| TGF-beta signaling pathway |  | X |
| Taurine and hypotaurine metabolism |  | X |
| Terpenoid backbone biosynthesis |  | X |
| Tetracycline biosynthesis |  |  |
| Thiamine metabolism |  | X |
| Tight junction |  | X |
| Toluene degradation |  | X |
| Toxoplasmosis |  | X |
| Transcription factors |  | X |
| Transcription machinery |  | X |
| Transcription related proteins |  | X |
| Translation factors |  | X |
| Translation proteins |  | X |
| Transporters |  | X |
| Tropane, piperidine and pyridine alkaloid biosynthesis |  | X |
| Tryptophan metabolism |  | X |
| Tuberculosis |  | X |
| Two-component system |  | X |
| Type I diabetes mellitus |  | X |
| Type II diabetes mellitus |  | X |
| Tyrosine metabolism |  | X |
| Ubiquinone and other terpenoid-quinone biosynthesis |  |  |
| Ubiquitin system |  | X |
| VEGF signaling pathway |  | X |
| Valine, leucine and isoleucine biosynthesis |  |  |
| Valine, leucine and isoleucine degradation |  |  |
| Various types of N-glycan biosynthesis |  | X |
| Vascular smooth muscle contraction |  |  |
| Vasopressin-regulated water reabsorption |  | X |
| Vibrio cholerae infection |  | X |
| Vibrio cholerae pathogenic cycle |  | X |
| Viral myocarditis |  |  |
| Vitamin B6 metabolism |  | X |
| Wnt signaling pathway |  | X |
| Xylene degradation |  | X |
| Zeatin biosynthesis |  | X |
| alpha-Linolenic acid metabolism |  | X |
| beta-Alanine metabolism |  | X |
| beta-Lactam resistance |  | X |
| mRNA surveillance pathway |  | X |
| mTOR signaling pathway |  |  |
| p53 signaling pathway |  |  |

**Table S6.** **OTU Richness by Location.** OTU richness for *D. albipictus, D. occidentalis*, and *I. pacificus* larval microbiomes collected from China Camp State Park (CCSP) in Marin, CA, or Jack London State Park (JLSP) in Sonoma, CA. Values listed denote the mean and 1 standard deviation. No significant differences were detected between regions for any species using the Kruskal-Wallis test.

|  | ***D. albipictus*** | ***D. occidentalis*** | ***I. pacificus*** | **All Species** |
| --- | --- | --- | --- | --- |
| CCSP (Marin) | 19.40 ± 1.67 | 22.67 ± 1.75 | 25.5 ± 2.67 | 23.00 ± 3.27 |
| JLSP (Sonoma) | 21.0 ± 3.46 | 21.25 ± 5.12 | 27.0 ± 4.97 | 23.69 ± 5.23 |

**Table S7.** **OTU Diversity by Location.** OTU Shannon’s diversity for *D. albipictus, D. occidentalis*, and *I. pacificus* larval microbiomes collected from China Camp State Park (CCSP) in Marin, CA, or Jack London State Park (JLSP) in Sonoma, CA. Values listed denote the mean and 1 standard deviation. Statistical significance is determined via Kruskal-Wallis test, with (*) denoting a p-value < 0.05.

|  | ***D. albipictus*** | ***D. occidentalis*** | ***I. pacificus*** | **All Species** |
| --- | --- | --- | --- | --- |
| CCSP (Marin) | 1.93 ± 0.43 | 1.39 ± 0.87 | 1.37 ± 0.56 | 1.51 ± 0.67 |
| JLSP (Sonoma) | 1.33 ± 0.78 | 2.15 ± 0.14 | 1.33 ± 0.55 | 1.74 ± 0.65 |

**Figure S1.** Microbiome representation of nymphs by species. (a) Weighted and (b) Unweighted non-metric multidimensional scaling (NMDS) by species at the nymph life stage. Ellipses represent a 95% confidence interval around the centroid of each group.

**Figure S2.** Microbiome representation of adults by species. (a) Weighted and (b) Unweighted non-metric multidimensional scaling (NMDS) by species at the adult life stage. Ellipses represent a 95% confidence interval around the centroid of each group.

**Figure S3.** Relative abundance of core microbiota by tick species. Abundance here refers to the percentage of sequence reads attributed to those OTUs identified as ‘core microbiota’ (See Methods: Core microbiome analysis) for each tick species.

**Figure S4.** Relative abundance of rare genera (See Methods: Sequence processing) by tick species. Abundance here refers to the percentage of sequence reads attributed to all OTUs present at less than 1% in all sequenced samples.

**Figure S5.** Microbiome composition by tick species and adult sex. A representative, or averaged, microbiome is shown for each tick species with all life stages included. Colors represent OTUs at the genus level, and bar heights corresponds to OTU relative abundance as determined by the percentage of sequence reads.

**Figure S6.** Microbiome representation of larval *D. albipictus, D. occidentalis,* and *I. pacificus* by region (China Camp State Park in Marin, CA or Jack London State Park in Sonoma, CA). (a) Weighted and (b) Unweighted non-metric multidimensional scaling (NMDS) by species and region at the larval life stage. Ellipses represent a 95% confidence interval around the centroid of each group.
